# Federated Learning in Computational Toxicology: An Industrial Perspective on the Effiris Hackathon

**DOI:** 10.1021/acs.chemrestox.3c00137

**Published:** 2023-08-16

**Authors:** Davide Bassani, Alessandro Brigo, Andrea Andrews-Morger

**Affiliations:** Pharmaceutical Research & Early Development, Roche Innovation Center Basel, F. Hoffmann-La Roche Ltd., 4070 Basel, Switzerland

## Abstract

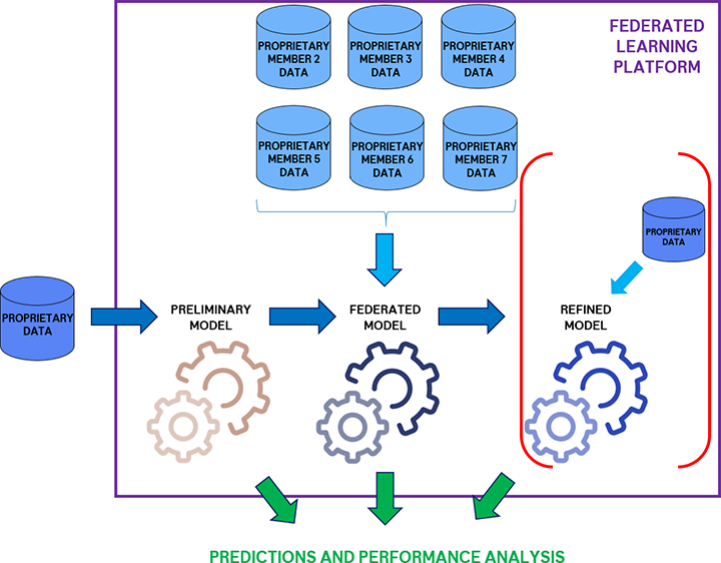

*In silico* approaches have acquired a towering role in pharmaceutical research and development, allowing laboratories all around the world to design, create, and optimize novel molecular entities with unprecedented efficiency. From a toxicological perspective, computational methods have guided the choices of medicinal chemists toward compounds displaying improved safety profiles. Even if the recent advances in the field are significant, many challenges remain active in the on-target and off-target prediction fields. Machine learning methods have shown their ability to identify molecules with safety concerns. However, they strongly depend on the abundance and diversity of data used for their training. Sharing such information among pharmaceutical companies remains extremely limited due to confidentiality reasons, but in this scenario, a recent concept named “federated learning” can help overcome such concerns. Within this framework, it is possible for companies to contribute to the training of common machine learning algorithms, using, but not sharing, their proprietary data. Very recently, Lhasa Limited organized a hackathon involving several industrial partners in order to assess the performance of their federated learning platform, called “Effiris”. In this paper, we share our experience as Roche in participating in such an event, evaluating the performance of the federated algorithms and comparing them with those coming from our in-house-only machine learning models. Our aim is to highlight the advantages of federated learning and its intrinsic limitations and also suggest some points for potential improvements in the method.

## 1 Introduction

In the early 1960s, the idea that, by exploiting the same principles of human teaching and learning, it would have been possible to proficiently train a machine to suggest solutions for a given problem, started to spread in the scientific community.^1^ Nowadays, more than 60 years later, we can witness the revolution that Machine Learning (ML) brought to our everyday lives, being implemented in fields such as image recognition, life sciences, targeted advertisement, finance-related predictions, etc.^2−10^ The scientific world has greatly benefited from the implementation of these approaches, which allowed an unprecedented level of automation in various decision and production processes to be reached.^11,12^ In pharmaceutical development, ML techniques are routinely used for tasks such as molecular property prediction, classification of compounds based on one or more desired end points (e.g., activity, phototoxicity, passive permeability, etc.), and in the most recent years, *de novo* generation of chemical structures.^13−15^

In the field of ML, each object (which can be both concrete, like a molecule, a person, a building, etc., and abstract, such as a study, a behavior, an idea, etc.) can be represented as an independent entry, characterized by one or more “features”. These are the properties of the data point, which can be used by the algorithm to be educated and to learn as much as possible about the data point itself. Each data point is associated with a “label”, which identifies the property of interest in the ML engine. In the case of a classification task, the label will be a discrete class (numerical or nominal with more than one category), while in the case of a regression problem, the label is continuous.

An ML task starts typically with a process called “training”, during which the algorithm explores the correlations between the features of each data point and its label. From a practical perspective, this is translated into the progressive optimization of a so-called “loss function”,^16^ which ideally should decrease in a gradient-like fashion with the progression of the training process. The “knowledge” gained is then retained by the model and used in order to predict the label of new data points. Nevertheless, before using a trained model for predictions on unlabeled data, it is crucial to assess its performance on a so-called “validation set”, which is a group of data points of which the label is known but is not provided to the algorithm. The ML engine will predict the label of these entries, and the comparison between the predicted and the actual values can be used for the evaluation of the model performance.^17^ The main metrics that are adopted for this purpose strongly depend on the kind of outcome. In binary classifications, for example, the main values that are considered are the sensitivity (also known as “true positive rate”), specificity (or “true negative rate”), balanced accuracy, and the Matthews Correlation Coefficient (MCC).^18^ In a regression setting, the main metrics are related to a continuous independent variable prediction, such as correlation coefficient between the predicted and the actual labels (indicated as *R*^2^) or the mean absolute error (MAE).^19^

Over the years, many different ML architectures have been developed and optimized, from the more simple Linear and Logistic Regression models, to the widely used tree-based techniques (such as Random Forest, ExtraTrees, and Gradient Boosting), to other peculiar structures such as XGBoost or Ridge methods.^20^ Most recently, the recurrent availability of “big data” pushed the success of architectures such as complex Artificial Neural Networks, which have been demonstrated to be the gold standard when it comes to dealing with huge data sets.^21^ Moreover, when temporal and ordinal features play a relevant role in a prediction process, schemes such as Recurrent Neural Networks (RNNs), and more specifically Long–Short-Term Memory (LSTM) networks, were implemented with great success in many applications.^22^

In the small-molecule drug development scenario,^23−26^ where ML is applied mainly to predict a desired chemical, pharmaceutical, or physical property, the data points represent the chemical compounds themselves. These molecules are encoded in a series of features, which can span from the simple physicochemical descriptors to more complex representations such as pharmacophore fingerprints^27,28^ or molecular graphs.^29^ The desired outcome may be a class (e.g., toxic/nontoxic, active/inactive, permeable/nonpermeable) or a continuous property (e.g., octanol–water partition coefficient, probability of on-target toxicity,^30^ fraction distributed in tissue, likelihood of toxicity in clinical trials,^31^ etc.).

### 1.1 The Federated Learning Approach

One challenge of applying ML in the drug development process is the availability of sufficient quantities of high-quality data.^32,33^ Indeed, this is crucial in order to build reliable and consistent models with a “virtual knowledge” that is comprehensive enough to be generalized to a vast space of unlabeled data points. The amount of data required for this task is very dependent on the final purpose of the algorithm and on its desired applicability domain.^34−36^

Usually, big data come from very large and/or complex databases, which can be both publicly available and privately owned. As it is well-known, public data has the great advantage of being more easily collectible in high quantities, but of course, their cleanness and reliability is much less pronounced compared to private data, which are expected to be of the highest quality but at the price of being confined to a specific space (e.g., a company interest) or simply numerically limited.^37^ Big data sets can be built by combining the information on several data sets, and ideally, the best data-oriented solution would be to create merged and comprehensive data sets from private sets, in which an agreement on how to collect and organize the data would have to be in place. When it comes to research, this is not feasible because of the obvious issues related to confidentiality and intellectual property protection. This is also valid in the drug discovery context, where the physical, chemical, and functional properties associated with small molecules are essential to a company investing significant resources in their development. So, the main question is how to get some information from all these private data sets without necessarily sharing sensible data among participating companies. One approach to utilize data stored at and owned by different companies without sharing any sensitive data is federated learning.^38^ This specific framework consists of the training of an algorithm via multiple independent sessions, each dependent on its own data set. In this fashion, machine learning is enabled over non-collocated data, distributing the learning effort among different devices and making it possible to share knowledge among companies or individuals without confidential data disclosure.^39^ The so-created “local models” are then used for the creation of a unique “global model”, which comes from the combination of the parameters used by the local ones. For example, when dealing with neural network architectures (which are very common nowadays in the machine learning scenario), this is translated into the exchange of the “weight” and “bias” parameters characterizing the local neural network models with the global one. The advantage of this strategy is that the model parameters, which come from the training with confidential data but do not carry explicit information about them, can be transferred and exchanged for the improvement of a global model, which benefits from all the “knowledge” acquired by the local ones.^40^

Federated learning has already been applied to drug discovery, as assessed by the recent outcomes of the MELLODDY project, which involved ten pharma companies, sharing federated information for a total of more than 21 million small molecules.^41,42^ The MELLODDY approach uses multitask learning and underlying deep neural network architectures. In brief, local models were trained at each company, and only the gradients were exchanged, thus avoiding the disclosure of information on the underlying data and on the modeled end points.

In the present study, an alternative federated learning approach is employed,^43^ where the general idea is that the local models (teachers) are used to make predictions on a large unlabeled data set and that the federated model (student) will learn from the teachers as it is trained on the consolidated predicted labels^60^ generated by the local models.

In the present contribution, we present the Roche evaluation as participants in a federated learning hackathon organized by the Lhasa Limited company (Granary Wharf House, 2 Canal Wharf, Holbeck, Leeds LS11 5PS, United Kingdom, which we will refer to as “Lhasa” from now on) by exploiting a commercial tool for secondary pharmacology prediction developed from them, called Effiris.^44^ This event involved the participation of seven different companies, all of which contributed to the building of the final federated model. Our focus will be centered on the validation results obtained with our proprietary data.

## 2 Experimental Procedures

Effiris is an ML federated platform developed by Lhasa to predict the on-target activity of small molecules based on their SMILES representation. The Effiris engine consists of an initial “classical” model-building step, in which the labeled data coming from the user are exploited for the prediction of a defined test set. The created model is then used in order to make predictions on another bigger, but unlabeled, set of points in a way that the knowledge acquired from the first model can then be transferred to the second one.

**Figure 1:**
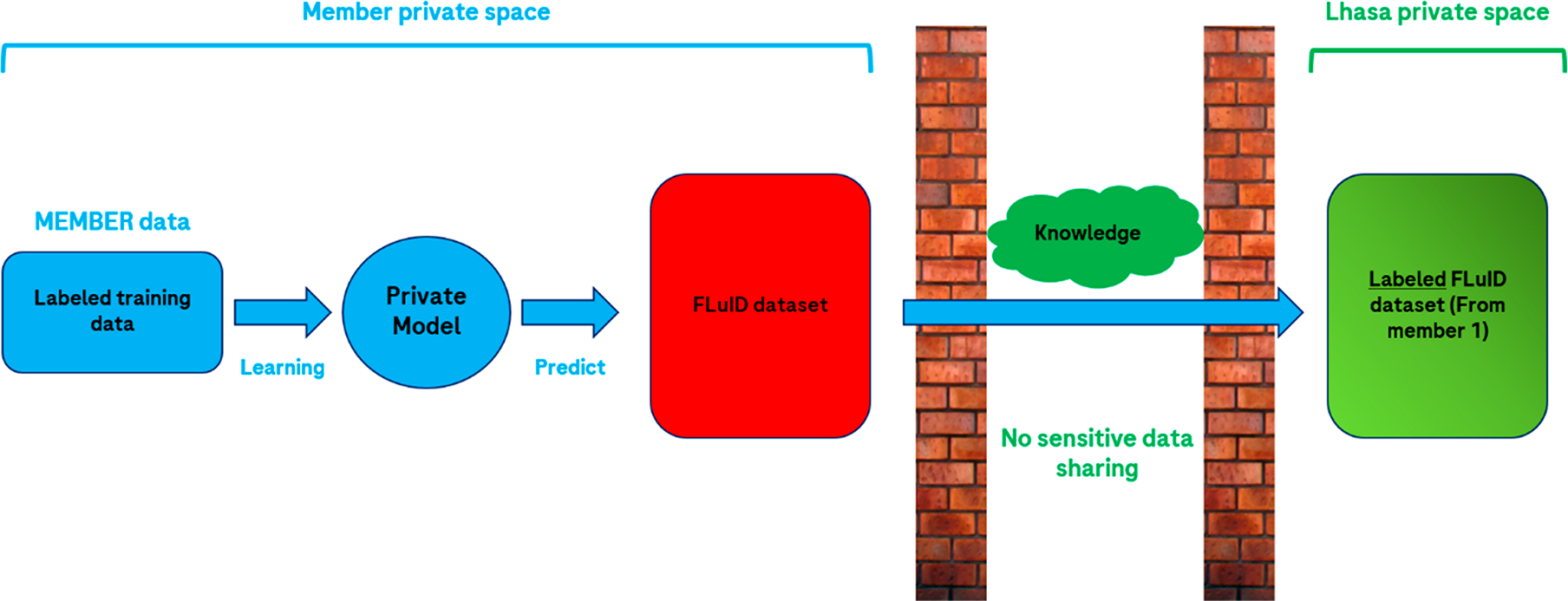
Representation of the main Effiris federated learning scheme for each of the company members.

The federated approach consists of multiple partners joining a “consortium”, and each partner is asked to provide the labeled data for the initial model building. This sharing effort comes with firewall protection in a way that no sensitive information can be seen, taken, or used by the consortium manager or by the other partners. In Effiris, the models made available for the hackathon were all based on binary classification, with the program giving just the binary outcome of the prediction. Within the platform, each user is asked to upload a defined set of compounds, each labeled with a positive or negative experimental activity marker for a defined biological target of interest. The operative engine of the Effiris platform is represented in Figure 1.

### 2.1 The Effiris Concept

**Figure 2:**
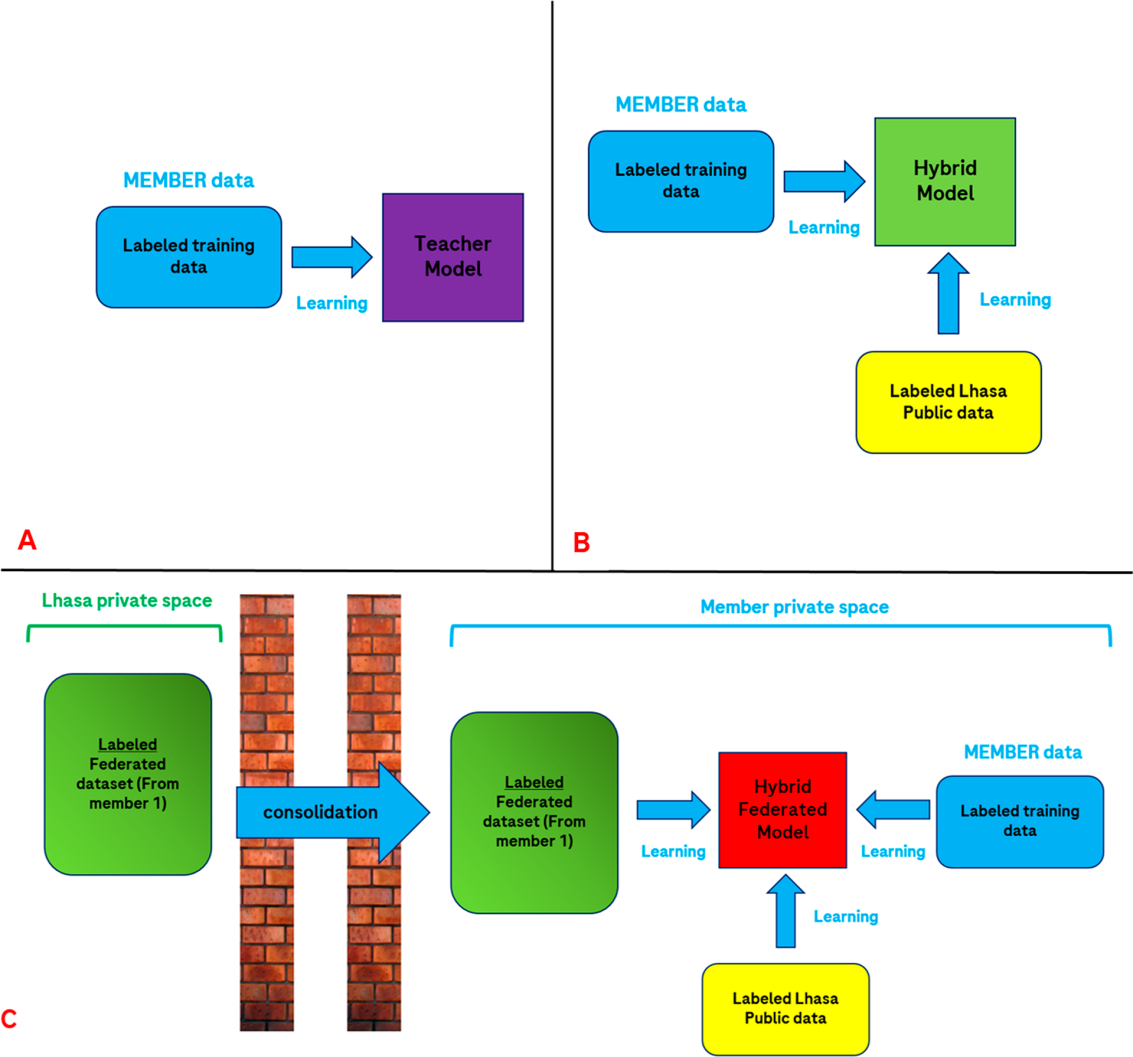
Representation of the Effiris SOHN federated learning scheme. The internal member data are used to train the “teacher” model (A), while both internal and Lhasa public data are combined to train the “hybrid” model (B). The final “hybrid federated” model is trained on the combination of internal, public, and federated data (C).

**Figure 3:**
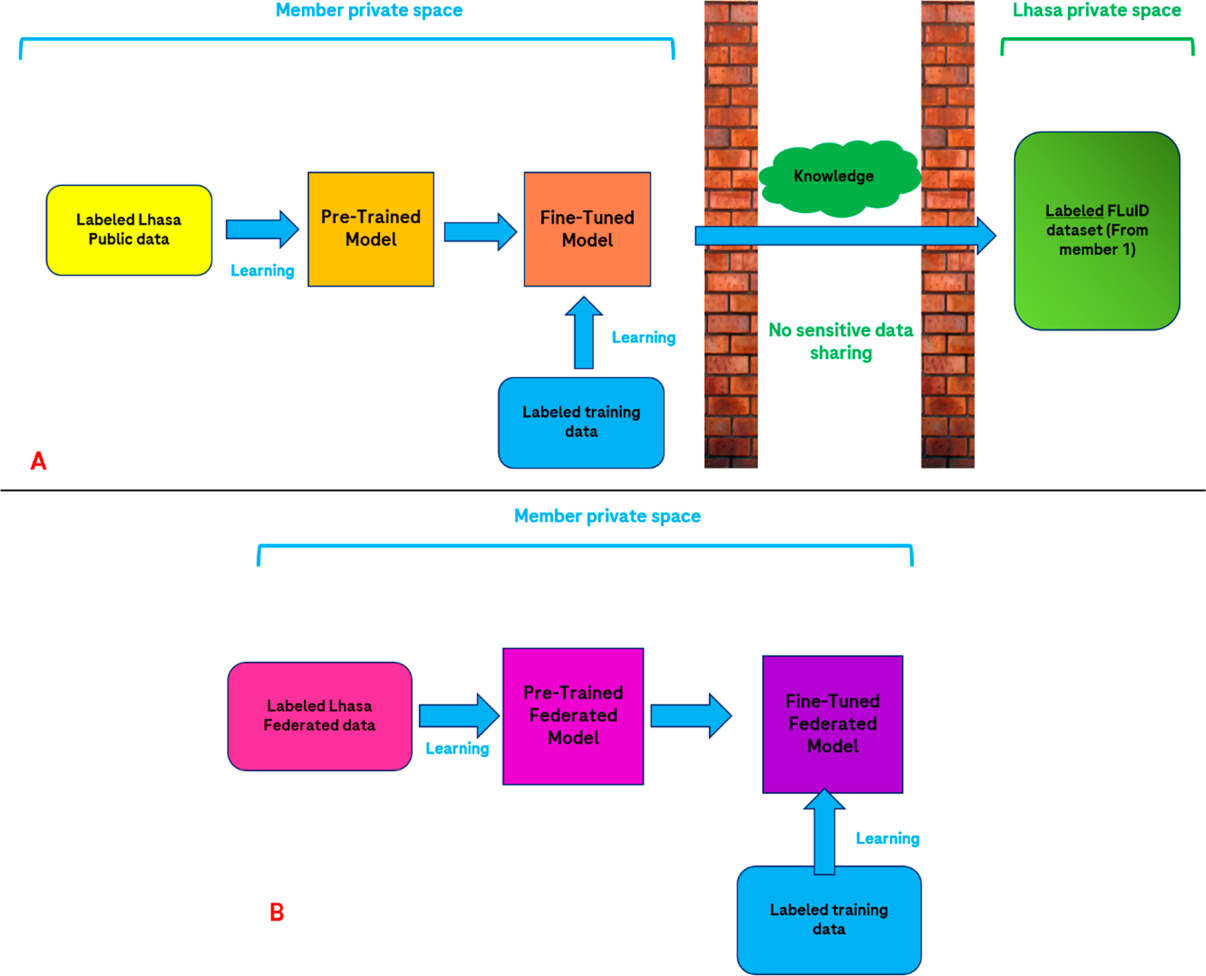
Representation of the Effiris MLP federated learning scheme. (A) An initial model is trained on public data curated by Lhasa. This pretrained model is then refined with internal data in order to get the “fine-tuned” MLP. (B) The federated labels obtained from predictions of consolidated “fine-tuned models” on the FLuID data from the members are then used to train another model, called “pretrained federated”, which is further refined with member data, generating the “fine-tuned federated” model.

When the partner data are uploaded, Effiris offers two main architectures for model building, which are the Self-Organizing Hypothesis Network (SOHN, Figure 2) and the Multilayer Perceptron (MLP, Figure 3). The technical details about their specific features are described elsewhere.^45,46^

Once the models have been trained on the data supplied by the different members in the hackathon, the knowledge acquired is used to predict the labels of a pool of more than 300,000 unlabeled, untested molecules, also called the “FLuID (Federate Learning using Information Distillation) data set”, which were sampled from a very large pool of public data by the Lhasa team. After the predictions on the FLuID data set are returned to Lhasa, they are consolidated by combining with the predictions from all members. Effiris provides two prediction attributes; the first is “Reliability” linked to the gap in chemical space between the molecules seen by the models in the training steps and the ones being labeled, and the second is “Decidability” related to the confidence in the prediction. After consolidation, the FLuID data set is filtered by Reliability (discarding all the predictions presenting a value below 0.005 for these metrics) and ranked by decreasing Decidability. The first 10,000 entries presenting a positive label, together with the 10,000 showing a negative one, are then merged in order to form the so-called “federated set”, which is the one then used for the training in case Effiris has to execute predictions on unlabeled molecules. In case one of the two classes does not reach 10,000 data points, the missing number of molecules is filled with compounds coming from the other class, always following the Decidability ranking.

These numbers were chosen by the Lhasa team according to the computational resources available for the algorithm training, in order to guarantee at the same time a big enough set for the training (formed by 20,000 compounds) and the positive/negative balance (ideally 1:1) within the set itself. The trained models can then be further refined in a user-defined fashion. In federated learning, with “model refinement”, we mean a fine-tuning of the federated model on another data set, which could be the local member training set. In this way, the global model, after having gathered the knowledge from all the members’ local models, is focused on the training set of a single member, refining its parameters further toward the local data. The participants can indeed choose to refine the Lhasa models (for now, only the MLP architecture) on a self-provided data set (e.g., the internal one) or on a Lhasa-provided set formed by compounds in which both structure and labels come from the literature (the details of these data sets are provided in Table 1 below and in greater detail in Table S1) or to not execute any refinement.

**Table 1:** Numerical Composition of the Different Data Sets Considered in the Present Study

target	Roche training set (inactive/active)	Roche test set (inactive/active)	SOHN Federated set (inactive/active)	MLP Federated set (inactive/active)	Lhasa Public set (inactive/active)
AChM_1_R	3811 (2829/982)	876 (745/131)	20,000 (10,000/10,000)	20,000 (10,000/10,000)	2191 (1288/903)
GABA_A_	3614 (3216/398)	877 (819/58)	20,000 (10,000/10,000)	20,000 (14,915/5085)	2433 (1645/788)
5-HT_2B_	3374 (2447/927)	876 (709/167)	20,000 (10,000/10,000)	20,000 (10,000/10,000)	1506(685/821)
hERG	2285 (1388/897)	556 (309/247)	20,000 (10,000/10,000)	20,000 (10,000/10,000)	8298 (4157/4141)
COX2	3042 (2815/227)	881 (809/72)	20,000 (12,233/7767)	20,000 (10,535/9465)	3228 (1545/1683)

### 2.2 Data Collection and Analysis

#### 2.2.1 Internal Data Gathering

Among the available targets, each hackathon member was asked to choose a defined pool and prepare a data set of in-house compounds labeled with respect to their activity for those specific targets. Our analysis focused on five targets for ML model building, which were “Cyclooxygenase-2 inhibition” (also known as COX2), “GABA_A_ receptor binding” (specifically, in the benzodiazepine binding site), “hERG channel inhibition”, “Muscarinic acetylcholine receptor M_1_ binding” (AChM_1_R), and “Serotonin 2B (5-HT_2B_) receptor binding.

For each of these five targets, we collected in-house experimental data to form five independent data sets. As defined by the Effiris consortium, compounds were labeled as active (indicated with 1) if the percentage of binding or inhibition was equal to or greater than 50% at a fixed concentration of 10 μM, while measurements below 50% were marked as “inactive” (indicated with 0). For a temporal validation of the predictive performance of the models, each data set was divided into training and test data. Molecules that were experimentally tested in 2020 or later were separated and kept as a test set, while everything tested before was used as a training set. With this approach, we simulate the behavior of a model that, in a real case scenario, would have to make predictions on progressively more recent molecules with expanded chemical space. The numerical compositions of the compound set created within Roche are represented in Table 1 in the following section.

#### 2.2.2 Overall Data Description

In Table 1, the numerical composition of the different data sets used in the present study is reported. Besides the already mentioned internal Roche training and test sets, we also report the exact number of active and inactive molecules included in each federated data set and also in the data set of public compounds that were gathered by Lhasa for the training of their own algorithms.

#### 2.2.3 Analysis of the Data Set Balance

Before evaluating the predictive performance of the algorithms, an important aspect to consider is the negative/positive ratio in the training and test data sets used. The Lhasa federated data set is artificially built to be balanced, and the Lhasa public sets were gathered and collected in a way that this balance is maintained (the negative/positive ratios range from 0.83 for 5-HT_2B_ up to 2.09 for GABA_A_). On the other hand, the internal training data sets show negative/positive ratios ranging from 1.55 for hERG to up to 10.16 for COX2, while for the test sets the ratios span from 1.25 for hERG up to 14.12 for the GABA_A_ receptor binding. Moreover, even the federated data sets are not always strictly balanced. Indeed, in the case of GABA_A_, the MLP predictions on the FLuID data set presented only 5085 positive entries, and these were all eligible in terms of Decidability and Reliability to become federated labels. For this reason, this particular federated data set includes 14,915 negatives instead of 10,000, and so, the negative to positive ratio raised from the ideal value of 1.00 up to 2.93. The same is true for the predictions executed on the FLuID data sets for COX2 from both MLP and SOHN models. In the first case, the number of eligible positive labels is 9465, causing the negatives to be 10,535 (and, consequently, the negative/positive ratio to be 1.11), while in the second scenario, the eligible positives were 7767, determining an increase in the negatives to 12,233 (and the ratio to 1.58). In order to overcome these limitations, several techniques are available, among which one of the classical is the so-called “oversampling” approach,^47^ in which the minority labeled class in the data set is replicated by a certain discrete amount of times prior to the training, up to reaching a more “balanced” overall data set. Even if these methods have shown some potential in different ML studies,^48,49^ our internal efforts in their application with the data sets of interest for this work did not show any significant increase in the algorithm’s predictive performance. The main reason is that even if these methodologies can help the models to better identify the features discriminating positives from negatives, they cannot really increase the number of these attributes.

#### 2.2.4 Data Preparation

The SMILES string corresponding to the molecules were parsed through an in-house created python script, based on the RDKit Python library,^50^ in order to be processed and made suitable for ML. Specifically, all the salts were removed, keeping just the largest molecular fragment for each molecule, and the SMILES were canonicalized. For the generation of the internal Roche data sets, the whole pool of 1D and 2D physicochemical descriptors (for a total of 208 physicochemical features) from the RDKit package were calculated for each molecule, together with the Morgan fingerprints,^51^ which were encoded in 2048-bit vectors, taking into account a radius of 3 bonds. Each of the descriptors was then scaled in a numerical interval from 0 to 1 (by exploiting the MinMaxScaler function implemented in the Scikit-learn^52^ python package), in order to avoid that some could prevaricate on others during the model training just for their different nature. To be more rigorous, we executed the calculation and scaling with the training and test set together in order to avoid undesired unconformities in the scaling processes between the two. Of course, after this step, the test set was unequivocally separated from the training set.

#### 2.2.5 Chemical Space Analysis

One of the main aspects in predicting molecular properties relies on the coverage of the chemical space that the training set can guarantee, together with the difference between the training and test sets’ chemical spaces.^53^

For an analysis of the chemical space covered by the data sets, we implemented the t-Distributed Stochastic Neighbor Embedding (t-SNE) method.^54^ Each t-SNE plot was built on the same features on which the ML models were trained, considering the whole pool of the 1D and 2D physicochemical descriptors of the RDKit package together with the 2048-bit Morgan fingerprints. For each representation, we reduced the complexity of the training set first to two dimensions and then to three dimensions.

**Figure 4:**
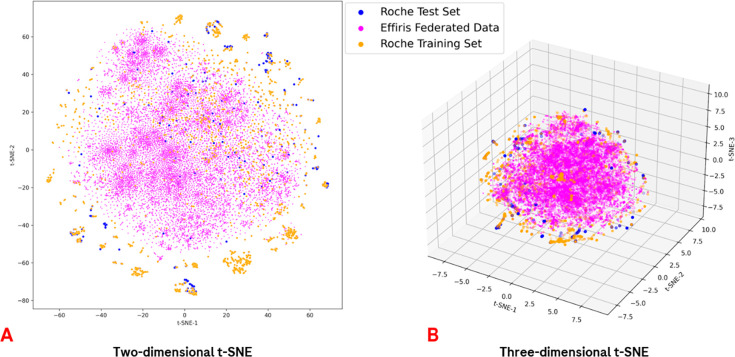
t-SNE plots representing the distribution of the chemical space for the AChM_1_R data sets. Specifically, panel A depicts the reduction of the pool of features composed by the 208 RDKit physicochemical descriptors and the 2048-bits fingerprints to just two dimensions, while in panel B, the three-dimensional reduction is reported.

One of the examples of the t-SNE-based chemical space analysis, focused on the AChM_1_R case, is reported in Figure 4, while for the other selected targets, the resulting plots are reported in the Supporting Information. As can be seen, the chemical spaces covered by the Lhasa federated data set are not strongly overlapping with the internal Roche training and test sets, with many points clustering outside the central portion of the plot. Nevertheless, the test set points are reasonably covered by the internal Roche training set. Even if similar behaviors can be found for the other targets, the specific t-SNE scheme is target dependent.

Nevertheless, in the overall picture, it seems that the federated data sets could be improved in terms of molecular diversity, allowing a higher degree of confidence in making predictions on a variety of different chemical series, which are expected to be variegated among the different partners and clients of the platform.

### 2.3 Internal Machine Learning Model Building

The in-house ML models were created by implementing the main classification architectures available in the Scikit-learn Python package. Specifically, the tested schemes were Gaussian Naïve-Bayes (GN), Steepest Gradient Descent Classifier (SGD), Decision Tree Classifier (DT), Random Forest Classifier (RF), ExtraTrees Classifier (ET), K-Nearest Neighbors Classifier (KNN), Multilayer Perceptron Classifier (MLP), AdaBoost Classifier (AB), Gradient Boosting Classifier (GB), and Support Vector Classifier (SVC).^20^ Moreover, also the eXtreme Gradient Boosting, or XGBoost (XG), algorithm was implemented.^55^ For each of the five targets of the hackathon, the training set was used by each of the algorithms in order to perform a hyperparameter optimization,^56^ which was based on a Stratified 5-Fold Bayes Search Cross Validation for 10 independent repeats. The hyperparameters that were prioritized for each model were scored on the balanced accuracy metrics and refitted on the sensitivity.

Once the hyperparameters were optimized, we executed both a stratified and a Random 5-Fold, 10-repeats Cross Validation for each model on the training set. Afterward, we used the optimized model in order to predict the labels for the test set (all compounds experimentally tested in 2020 or later).

### 2.4 Evaluation Metrics

**Figure 5:**
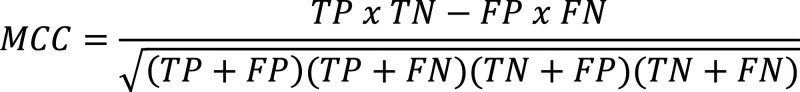
Mathematical formula for the Matthews Correlation Coefficient (MCC) calculation. The acronyms are the following: TP = True Positive; TN = True Negative; FP = False Positive; FN = False Negative).

For the evaluation and comparison of the predictive performance, the sensitivity, the specificity, the balanced accuracy (which is the arithmetic mean of specificity and sensitivity), and the Matthews Correlation Coefficient^57^ (also referred to as MCC, which is calculated with the formula reported here below in Figure 5 and ranges from −1 to 1) were used as metrics.

These metrics were then compared with those obtained both from Cross Validation (CV) and from the predictions on the test set to those from the Effiris models. In order to get a comprehensive overview of the impact of each type of data (internal, public, and federated) on the final predictions, we compared the performance of each ML setup.

We compared the Effiris models (SOHN and MLP) to our in-house built models. For SOHN, we present results for the models trained on federated data. For MLP, we will discuss both the models trained on federated data and those trained on federated data with Roche data refinement. For SOHN Lhasa models, the data was always combined with public data before the training, regardless of their initial training set, while for MLP, the algorithm pretraining was executed on the public data or on the public data combined with the federated data.

On the in-house side, the model trained on the best performing algorithm will be presented, also distinguishing the different data sets for their training. Namely, they are (1) the Roche data only; (2) the MLP Lhasa federated labels; (3) the MLP Lhasa federated labels together with Roche data; (4) the MLP FLuID labels; (5) the SOHN Lhasa federated labels; (6) the SOHN Lhasa federated labels together with Roche data; (7) the SOHN FLuID labels.

Precisely, the MLP and SOHN federated labels are the ones coming from the FLuID labels, after the filtration steps involving the Decidability and Reliability criteria (both data sets contain 20,000 predictions). The MLP and SOHN FLuID labels, on the other hand, include all 300,000 and more labels of the whole FLuID data set.

## 3 Results

In the following section, we provide an overview of the results obtained in our comparative study. First, we discuss the outcomes of the Cross Validation (CV) experiments executed both with the Lhasa SOHN model and with the in-house built ones. Second, we look into the applicability domain concept and how the federated learning is beneficial to this parameter. Lastly, we present the predictive performance of Lhasa and Roche models, trained with different sets, on the Roche external test set.

### 3.1 Cross Validation Results

Before comparing the predictive performance of the algorithms on the Roche test set, we performed CV experiments with both the internal and the Lhasa models, as it is common practice for ML. The CV allows one to obtain an idealistic upper bound for the predictive performance of the models on an external set, because it assesses the performance of the model if it is applied to data sets stemming from the same distribution as the training data (which actually is more hypothetical than realistic).

In the Effiris platform, only the SOHN model is able to perform a proper 5-fold random split CV on the uploaded data sets. In order to test the pure model performance for both Lhasa and internal models, we subjected to this experiment all of our five data sets and then compared the outcomes of both the best algorithm tested internally and the Lhasa ones. The results for the Roche models are presented in Tables 2 and 3 (stratified and random split, respectively), while the ones from Lhasa are shown in Table 4 (SOHN model). As can be seen, the top performing ML internal architectures when using the stratified approach are XGBoost and SVC, while in the case of the random split, the top performing model is the Random Forest Classifier. In both cases, the CV results appear to be slightly improved compared to the SOHN in Effiris for all of the targets considered. It is important to mention that only the random 5-fold approach (Table 3) is effectively comparable with the Lhasa results (Table 4). Indeed, the implementation of the stratification, which periodically keeps the same negative/positive ratio in both training and test sets for each of the folds, may help the model improve its performance. The implementation of a cluster-based Group K-fold CV, which is more robust for molecular properties prediction (it simulates the scenario in which the test set has the least resemblance to the training set),^58^ was not executed in this experiment because of the potential great difference with the random split used by the Lhasa platform. Indeed, the cluster-based CV would have made the outcomes even less comparable than those with the stratified approach. In any case, the overall CV outcomes are similar for Effiris and the internal models, indicating no obvious advantage in shifting from one architecture to another.

**Table 2:** Results for the Best-Performing Internal Model in the 10-Repeated, 5-Fold Stratified Cross Validation Experiment Executed on the Internal Data Sets^a^

target	model	balanced accuracy	specificity	sensitivity	precision	AUC	MCC
AChM_1_R	XGBoost	0.83 ± 0.01	0.95 ± 0.01	0.71 ± 0.03	0.83 ± 0.03	0.83 ± 0.01	0.69 ± 0.02
GABA_A_	SVC	0.84 ± 0.02	1.00 ± 0.00	0.69 ± 0.04	0.95 ± 0.02	0.84 ± 0.02	0.79 ± 0.03
COX2	XGBoost	0.67 ± 0.04	0.99 ± 0.01	0.35 ± 0.08	0.70 ± 0.10	0.67 ± 0.04	0.47 ± 0.08
hERG	SVC	0.78 ± 0.02	0.86 ± 0.02	0.70 ± 0.03	0.76 ± 0.02	0.78 ± 0.02	0.56 ± 0.03
5-HT_2B_	XGBoost	0.78 ± 0.02	0.87 ± 0.01	0.70 ± 0.03	0.66 ± 0.02	0.78 ± 0.02	0.56 ± 0.03

a The best outcomes are usually associated with the XGBoost and SVC architectures.

**Table 3:** Results for the Best-Performing Internal Model in the 10-Repeated, 5-Fold Random Cross Validation Experiment Executed on the Internal Data Sets^a^

target	model	balanced accuracy	specificity	sensitivity	precision	AUC	MCC
AChM_1_R	Random Forest	0.81 ± 0.01	0.97 ± 0.01	0.65 ± 0.03	0.89 ± 0.03	0.81 ± 0.01	0.69 ± 0.03
GABA_A_	Random Forest	0.84 ± 0.03	0.99 ± 0.00	0.68 ± 0.05	0.94 ± 0.03	0.84 ± 0.03	0.78 ± 0.04
COX2	XGBoost	0.66 ± 0.03	0.99 ± 0.00	0.33 ± 0.06	0.71 ± 0.08	0.66 ± 0.03	0.46 ± 0.06
hERG	Random Forest	0.78 ± 0.02	0.87 ± 0.02	0.69 ± 0.03	0.77 ± 0.03	0.78 ± 0.02	0.57 ± 0.03
5-HT_2B_	XGBoost	0.76 ± 0.02	0.92 ± 0.01	0.61 ± 0.03	0.74 ± 0.03	0.76 ± 0.02	0.56 ± 0.03

a The best outcomes are usually associated with Random Forest and XGBoost architectures.

**Table 4:** Outcomes of the Cross Validation Experiment Executed on the Internal Data Sets by Exploiting the SOHN Model Architecture Implemented in the Effiris Platform

target	model	balanced accuracy	specificity	sensitivity	precision	AUC	MCC
AChM_1_R	SOHN	0.83 ± 0.01	0.94 ± 0.01	0.72 ± 0.03	0.79 ± 0.03	0.90 ± 0.02	0.68 ± 0.02
GABA_A_	SOHN	0.87 ± 0.02	0.98 ± 0.01	0.77 ± 0.05	0.80 ± 0.03	0.94 ± 0.01	0.76 ± 0.04
COX2	SOHN	0.67 ± 0.02	0.97 ± 0.01	0.36 ± 0.03	0.55 ± 0.15	0.84 ± 0.02	0.40 ± 0.08
hERG	SOHN	0.75 ± 0.01	0.86 ± 0.02	0.64 ± 0.02	0.70 ± 0.03	0.82 ± 0.01	0.52 ± 0.02
5-HT_2B_	SOHN	0.75 ± 0.03	0.91 ± 0.07	0.58 ± 0.05	0.72 ± 0.03	0.85 ± 0.03	0.53 ± 0.05

### 3.2 The Applicability Domain (AD)

**Figure 6:**
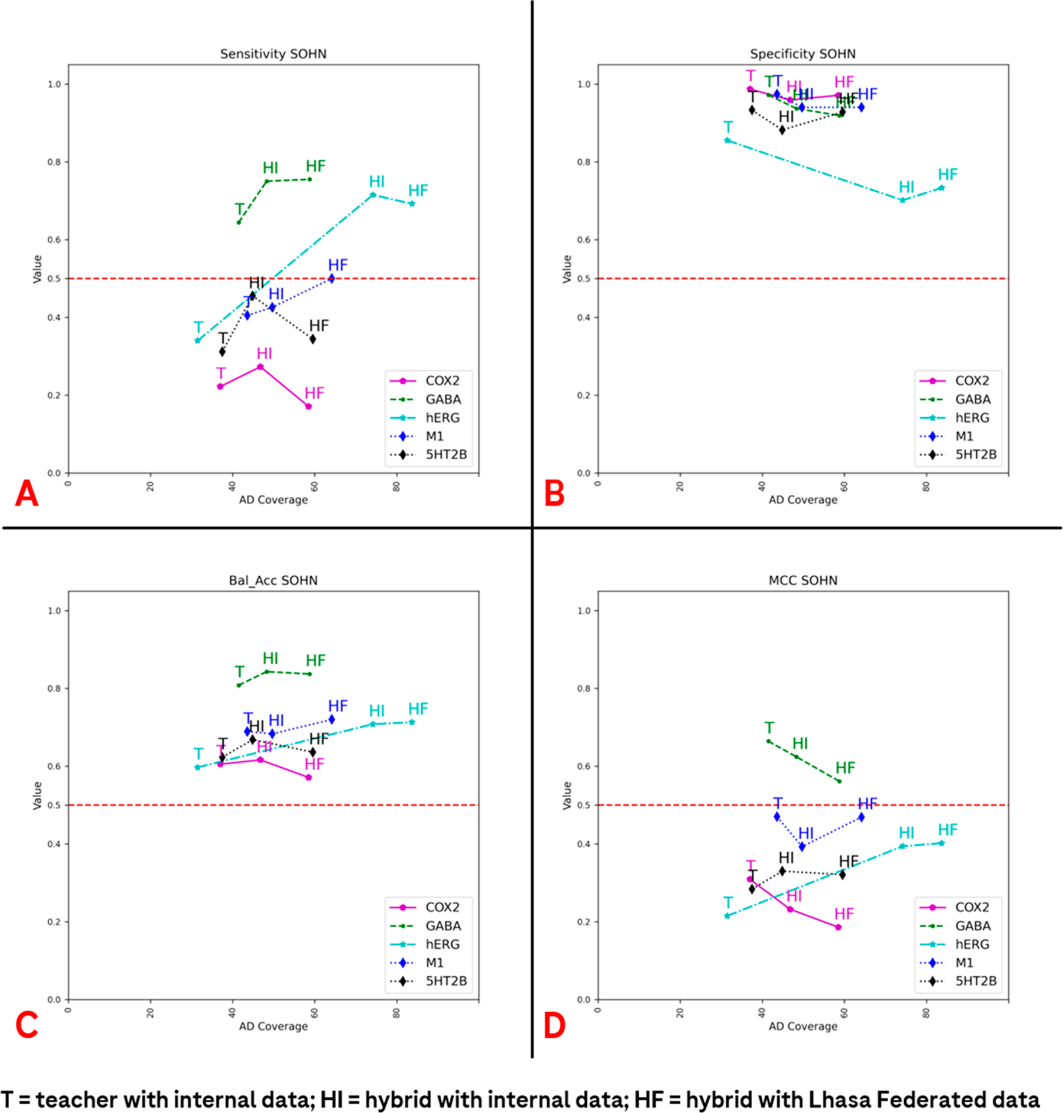
Trends in the Applicability Domain (AD) compared to the main performance metrics considered in the present study of the Lhasa SOHN architecture. The AD versus the sensitivity, specificity, balanced accuracy, and MCC of the prediction on the external test set are reported in (A–D), respectively. As the legend shows, the purple line indicates the data coming from the predictions on the COX2 data set, the green one stands for GABA_A_, the cyan for the hERG channel, the dashed blue line for AChM_1_R, and the dashed black line depicts the outcomes for 5-HT_2B_. The different data sets considered are also indicated with a different letter, as reported below. Specifically, T indicates metrics and AD for the “teacher” SOHN model (trained just on the internal Roche data); HI stands for “hybrid internal” and represents the SOHN model trained on both Lhasa public data and Roche internal data. Finally, the acronym “HF”, or “hybrid federated”, indicates the SOHN model trained on both federated data and Roche internal data.

One hypothesis around Effiris was that the added federated data could contribute to an increase in the applicability domain of the models. The Effiris platform offers an estimation of the applicability domain, which separates applicability, reliability, and decidability domains.^59^ The concept of the three domains is very nicely introduced by Hanser et al.^59^ In brief, the applicability domain determines whether a prediction is within the model’s descriptor, label, and structural feature space. The reliability of a prediction is defined based on the data quality, quantity, and relevance available to the model, and the decidability is determined based on probability or likelihood predictions, indicating whether a prediction is decisive or equivocal. Through the calculation of these values for each molecule, the platform allows one to calculate the percentage of the test set that can be predicted with a certain degree of confidence, which is also related to the chemical space covered by the training set. Intuitively, the bigger the training set and the larger its specific chemical space, the higher is the percentage of test molecules falling into the applicability domain. For each prediction round, Effiris offers the possibility to restrict the calculation only on the portion of the test set falling into the applicability domain or to ignore this parameter and execute predictions on the whole test set. When excluding the predictions for the molecules outside the AD, the performance of the Effiris algorithms are then evaluated only on the subset of the test set falling into the AD. In Figure 6, the percentage of predictions inside the AD is plotted versus other performance measures (sensitivity, specificity, balanced accuracy, and MCC) for the SOHN models.

It is noted that although the Reliability and Decidability metrics do not contain any structural information, there might be a small risk of privacy leakage, which is mitigated by several precautions: First, the end user as well as the aggregating party (i.e., Lhasa) have no access to the teacher model or any underlying training data. Second, only predictions on public data are shared with the aggregating company, and the convoluted data accessible to the end user cannot be traced back anymore. Third, even in the rare case that anything could be backtracked, this would reveal only information on public compounds.

**Figure 7:**
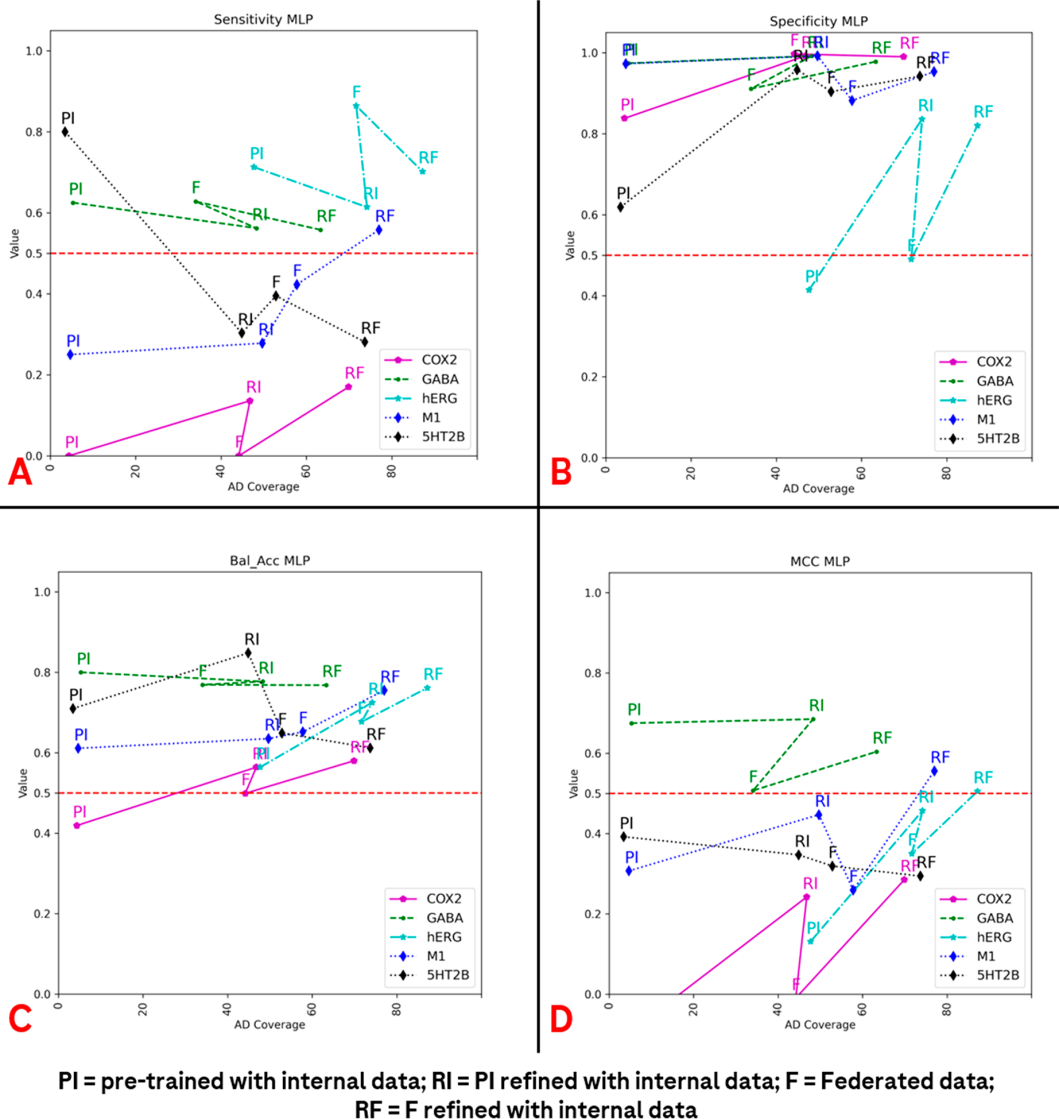
Trends in the Applicability Domain (AD) compared to the main performance metrics considered in the studies presented here for the Lhasa MLP architecture. Specifically, the AD versus the sensitivity, specificity, balanced accuracy, and MCC of the prediction on the external test set are reported in (A–D), respectively. As the legend represents, the purple line indicates the data coming from the predictions on the COX2 data set, the green one stands for GABA_A_, the cyan for the hERG channel, the dashed blue line for AChM_1_R, and the dashed black line depicts the outcomes for 5-HT_2B_. The different data sets considered are also indicated with a different letter, as reported below. Specifically, PI indicates metrics and AD for the pretrained internal MLP model (trained just on the Lhasa public data); RI stands for “refined internal” and represents the MLP model trained on Lhasa Limited public data and then refined with Roche internal data. Then, the letter “F”, or “federated”, indicates the MLP model trained on just the federated data, while “RF” (“refined federated”) represents the MLP trained on the federated data and then refined using Roche internal data.

Across all targets, an increase in percentage in domain predictions was observed from the “teacher” (T) to the “hybrid internal” (HI) and to the “hybrid federated” (HF) model. Figure 7 shows the AD vs performance change with the MLP algorithm when refining the “pretrained model on public data” (PI) or the “federated” (F) model, respectively. For every target, we could see an AD increase from PI to RI (which stands for “Refined with Internal data” and is indeed the PI model refined with internal data) and from F to RF (which is the F model refined with internal data). Likewise, the AD increased from RI to RF and from PI to F.

As can be seen, the general trend follows our previous observation both for the SOHN and MLP cases, with the applicability domain coverage increasing from the left to the right, indicating that the increase of the number of data points in the training set is beneficial for the increase in these metrics (it enlarges the chemical space in which the final predictions are confident enough). It is interesting to notice that, while for the SOHN this principle is valid for all the data sets, in the case of the MLP there are some exceptions. There are situations in which, passing from the internal (RI) to the federated (F) data, the applicability domain does not change significantly (e.g., COX2 and hERG) or even diminishes (e.g., GABA_A_). This indicates that, in these scenarios, the shift from the internal training set to the federated one does not give an advantage in terms of chemical space coverage with respect to the test set. This can be because the refinement with Roche data has a greater impact on the algorithm performance compared to simply increasing the number of training data points. In any case, with the final refinement on the Roche data, the AD always clearly increases, demonstrating that the federated data are beneficial on this parameter if added to the internal data.

### 3.3 Performance Comparison on the Roche Test Set: Effiris vs Roche Internal ML Models

#### 3.3.1 Effiris Model Performance

**Figure 8:**
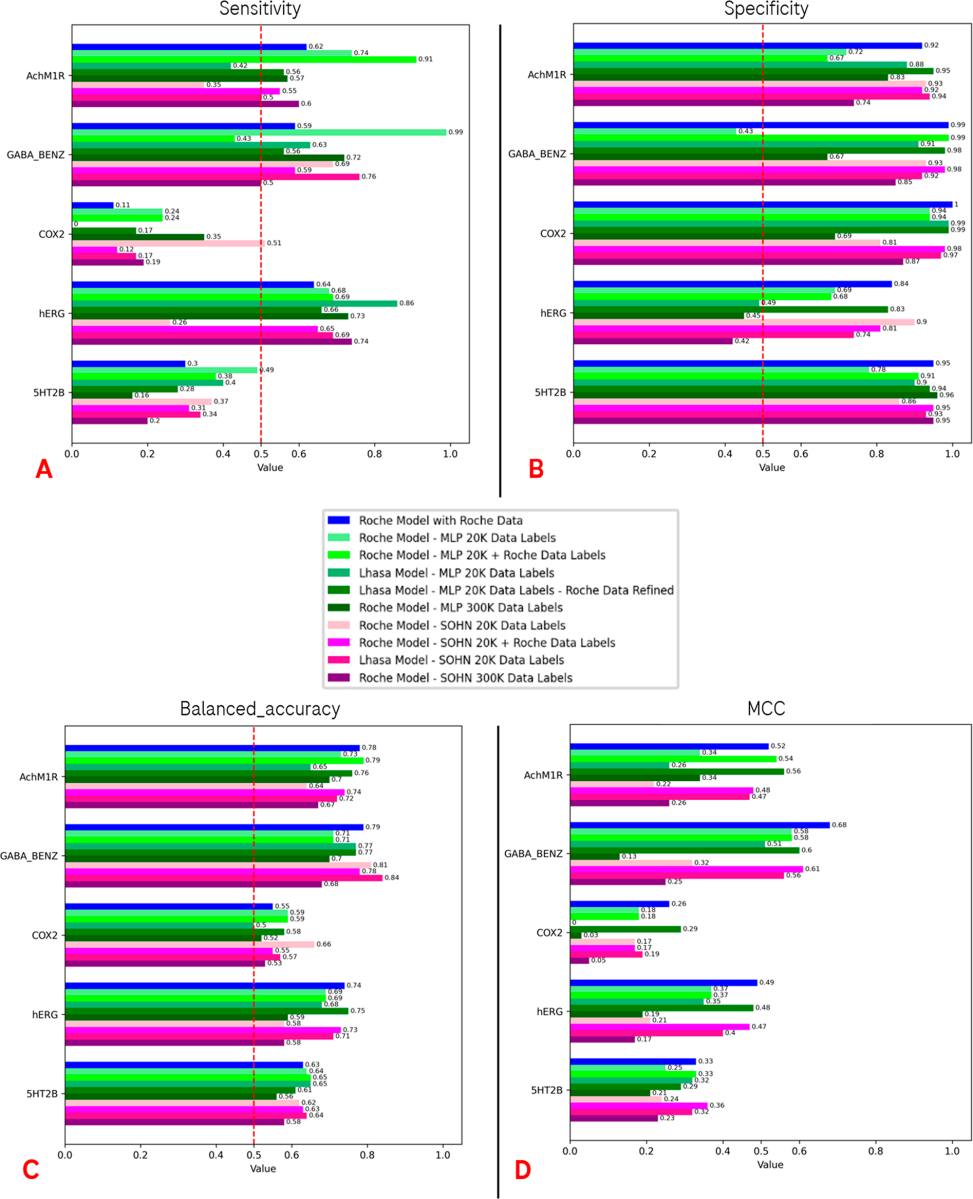
Plots representing the metrics of the predictions on the external test set, using the different training setups discussed in the previous paragraph (for both internal and Lhasa models). A–D represent the sensitivity, specificity, balanced accuracy, and MCC metrics for each of the cases considered, respectively. For the first three plots, also the 0.5 value is marked with a dashed red line, indicating the point below which the predictions start to lose relevance (half of the positives/negatives are misclassified by the algorithm).

For the predictions made with the Effiris platform, we considered only the ones excluding the points that are out of the applicability domain in order to only include the confident predictions in the evaluation. This choice was made in order to simulate a real-case application of the models in which only the predictions inside the AD are taken into account. As the Roche internal models (developed for this study) have no AD measure, all of the molecules in the test set were considered for the evaluation. It is also worth noting that the Lhasa models could not benefit from hyperparameter optimization due to the hackathon constraints to ensure a common setup across end points and participants. From the final results comparison, which is presented in Figure 8, we can see that the performance of the different models, other than being very target dependent, is not optimal for many of the targets.

Indeed, the MCC values obtained from both the SOHN and MLP models show that, while the SOHN algorithm trained on federated data (together with Roche and public data) always outperforms the MLP trained on the same data set, the best performance is obtained through the MLP algorithm trained on federated data and then refined on Roche data. The only exception to this trend is observed for the 5-HT_2B_ target, where the performance of MLP and SOHN trained on federated data are the same (MCC = 0.32), and the refinement with Roche data seems to slightly decrease the overall performance.

Looking at the other metrics considered in this study, for the Lhasa models, the trend is similar to the MCC. Concerning the balanced accuracy, the best algorithm tends to be the MLP trained on federated data and refined with internal data, but in this case, there are two exceptions, represented by 5-HT_2B_ and GABA_A_. In the first case, the MLP performance was the best without the refinement process, with the SOHN algorithm giving very similar results (Balanced_accuracy_MLP_ = 0.65 without refinement; Balanced_accuracy_SOHN_ = 0.64), while for GABA_A_, the SOHN outperformed MLP both with and without refinement (Balanced_accuracy_MLP_ = 0.77 both with and without refinement; Balanced_accuracy_SOHN_ = 0.84). In the case of GABA_A_, MCC is still higher for the refined MLP compared with SOHN (MCC_MLP_ = 0.60; MCC_SOHN_ = 0.56). Balanced accuracy is higher for the SOHN model (Balanced_accuracy_MLP_ = 0.77 both with and without refinement; Balanced_accuracy_SOHN_ = 0.84), and it is worth noticing that SOHN sensitivity performance is greater than both the MLP ones (Sensitivity_MLP_ = 0.63 without refinement; Sensitivity_MLP_ = 0.56 with refinement; Sensitivity_SOHN_ = 0.76); but, there is still a drop in specificity compared to the refined MLP (Specificity_MLP_ = 0.91 without refinement; Specificity_MLP_ = 0.98 with refinement; Specificity_SOHN_ = 0.92). The factor favoring the MCC_MLP_ with refinement with respect to MCC_SOHN_ in the case of GABA_A_ is probably related to the fact that the amount of inactive compounds in the GABA_A_ test set is much higher with respect to the active ones, so misclassifying them (as can be seen from the specificity) has a higher impact on the MCC value.

#### 3.3.2 Roche Internal Models Performance

In this section, we will provide an overview, divided by target, of the results obtained with the top-performing in-house model for each of the training sets that are described in Section 2.4. A panorama of the best algorithm in each scenario is reported in Table S2. As discussed in the previous paragraph, the results (represented in Figure 8) appear to be target dependent also for the ML models built internally. Even if, overall speaking, the performance is not optimal in the majority of the scenarios, in the case of GABA_A_, the model (SVC) performed quite better than the average, with an MCC value of 0.68.

Focusing mainly on the MCC as the more descriptive metrics for the performance of the algorithms, we can see that the Roche models trained only on internal data present the best performance on the test set, being the best performing internal models created in three out of five targets (GABA_A_, COX2, and hERG). Moreover, for GABA_A_ and hERG, this all Roche-based model setup was demonstrated to be the overall best performing for the target.

In the case of AChM_1_R and 5-HT_2B_, in which the Roche model built on Roche data is not the best among the ones we created internally, the internal model for which the Roche data were merged with the MLP federated labels for the training is the only one able to outperform and replicate the all Roche-based model performance. Nevertheless, it is important to highlight that these differences are not huge (0.02 for AChM_1_R and no difference for 5-HT_2B_).

Looking also at the balanced accuracy, AChM_1_R and hERG show a trend that is very similar to the MCC one, while for GABA_A_ and COX2, the best-performing models built internally come from the training just with the federated data obtained with the SOHN algorithm. For 5-HT_2B_, on the other hand, the best balanced accuracies come from the internal model trained on both the Roche data and the MLP federated labels, but also, in this case, the values are very similar to one another.

In any case, as discussed in the previous paragraph, the MCC results tend to be more informative than the simple arithmetic mean given by balanced accuracy, because this last value would not spot huge drops in sensitivity or specificity, while MCC also takes into account these eventualities.

#### 3.3.3 Effiris and Roche Internal Models in Comparison

Now that we have given a general overview of the performance of Effiris and Roche internal models separately, we discuss briefly how their performance compares. If we take into account the MCC values obtained with GABA_A_ (in which the Roche model trained just with internal data is the overall best performing across all settings) and hERG, we see that our all Roche-internal models were the top-performing for the target of interest. For AChM_1_R and COX2, even if the all-internal model was the runner-up in the predictive performance based on MCC, the best values were obtained with the Lhasa MLP model in which also the Roche data contribute to the refinement process (MCC_AChM1R_ = 0.56 and MCC_COX2_ = 0.29). Also, for the first two targets discussed, the Lhasa refined MLP model was among the best performing; i.e., as the second for hERG and the third for GABA_A_ (right after the SOHN model trained on both Roche and federated data) the Lhasa refined MLP models were also top performing.

The 5-HT_2B_ models, on the other hand, show behavior that is between the GABA_A_ and the COX2 ones. In this case, the Roche internal model performed best in terms of MCC, but the refinement with internal data of the Lhasa MLP model helps push the performance toward the same values. Moreover, the Roche model trained on the combination of federated and internal data outperformed the two already cited algorithms of 0.03 points in MCC.

Another aspect to consider is related to the inactive/active ratios in the data set examined. Indeed, while the federated sets are much more balanced (inactive/active ratios from 1.00 to 2.93) than the internal Roche data sets (inactive/active ratios from 1.55 to 12.40), the predictive performance tends to be higher when training on internal data. This is the result of the chemical space covered by the Roche compounds, which is closer to the one of the test set and is able to inform the models in a more proficient way compared to the federated data, despite the more distinct data imbalances.

Comparing Lhasa SOHN and MLP models with the Roche internally built models (trained on just Roche data, just federated data, or on the combination of the two), we can conclude that the best performance for the Lhasa models comes from the MLP algorithms refined on internal data, while in the case of our internal built models, federated data does not add to the model performance. The fact that, comparing the whole pool of algorithms in our experiment, the all-internal Roche models and the Lhasa MLP refined on Roche data perform the best (with a couple of exceptions in which the SOHN algorithm, with internal data implementation, is slightly top performing) is indicative that the presence of internal data in the training process is still key for the model performance.

Nevertheless, the performance of internal, SOHN, and MLP algorithms exploiting only federated data is in many cases promising, showing that the federated learning, even if still with some drawbacks, has potential in computational toxicology.

## 4 Discussion

### 4.1 Final Considerations on the Federated Learning Approach

In its overall scheme, federated learning offers the opportunity for different partners to get a predictive model based on the information obtained from the data coming from all of them, without effectively sharing sensitive data with one another and without compromising the confidentiality of the data. In order to see how much value this approach might add to the Roche internal workflows and pipeline, we participated in the Lhasa Hackathon and assessed the performance of the Effiris federated models, comparing them with in-house built ML models.

First, it is important to notice the increase in the AD, both for SOHN and MLP, passing from the models trained just on the Roche internal data set to the ones trained on the federated data. Such an increase in the AD with the help of federated learning has also been reported in the MELLODDY project.^41^ Furthermore, an additional AD increase is achievable when these latest models get refined with Roche internal data, i.e., in the case of MLP. Moreover, augmentation and continuous refinement of the FLuID data set, and hence the composition of the federated data, may lead to an even broader and more relevant coverage of the chemical space. The effect of the FLuID data set being more tailored toward drug-like molecules on the AD is expected to be 2-fold: First, the FLuID molecules will more likely be in the AD of the teacher models, making knowledge transfer smoother. Second, the federated training set is expected to have a higher coverage of internal molecules that can be predicted by the student model. This, together with the continuous and periodic supply of data from the different partners, is expected to allow the building of increasingly more stable and better performing teacher models, which would be able to transfer the knowledge acquired from the proprietary data to the federated model in a more efficient way.

It is envisaged that access to federated models would allow for more targets beyond those investigated during the hackathon. As the results of our study varied depending on the target investigated, it will be challenging to transfer learning from the evaluation for the five targets to other models available in Effiris. Nevertheless, availability to new models could be exploited both in a predictive manner *per se* and with an explorative approach, for example, if an industrial partner starts exploring activity on a toxicological target for the first time. In the latter scenario, together with the literature data, the federated models could be demonstrated to be helpful, allowing one to take advantage of the information already transferred by other industrial partners into the federated learning engine.

There are also some drawbacks and points that may be improved in this approach. The first limitation is related to the aspect that the federated data used to train the federated model consist of predictions rather than experimental labels. This introduces another level of uncertainty. While it is sometimes hard to get reasonable performance for the underlying teacher models trained on labeled data, models trained on the predicted labels are expected to be less reliable. It is noted that this may to some extent be further mitigated as proprietary data are added to the federated data when training the federated model (SOHN) or used to refine it (MLP), respectively.

Another very important aspect to consider is the fact that, for this approach to work, every partner should regularly provide the consortium with new data points, and their amounts should be reasonably proportional to the size of the company. As we have experienced, in our internal evaluation, the predictive performance usually did not improve by exploiting the federated models versus the internally built ones, and even if there was some slight improvement (which was still not enough to guarantee reliable predictions), this was achieved only through the refinement of the ML architecture on the internal Roche data. Given that the refinement with internal data seems to be important for the predictive performance, this raises the question of how reliably could the predictions be executed on a data set for a totally new target, in which there is no internal data available for the refinement process. Of course, other partners can provide such information to the platform, and in this scenario, the diversity in the chemical space of the federated set gains even more relevance, with this data set having the key role of transferring knowledge among the partners within the federated workflow.

An additional point has to be raised with respect to the model type implemented in Effiris. Indeed, in the current version, only classification models can be built, while regression models would be highly desirable when it comes to the optimization of on-target and off-target activities. Indeed, regression models can give more precise information about a specific molecular scaffold or structural pattern, guiding the design of further chemical entities toward a desired pharmacological and toxicological profile. Nevertheless, the platform is currently being extended to support regression in the future.

It is furthermore important to consider the experimental setup and conditions under which the data sets supplied from the different partners were produced. In a federated learning approach, all data points coming from the industrial members are reasonably equal, but if the assay conditions (temperature, pH, sampling, etc.) change between companies, this would, of course, affect the results of the models. Federated learning has no control over these variations. If the platform is developed further to also include regression models, this aspect would be even more challenging, given that the uncertainties brought by different partners’ measurements would impact even more the model in the training process. This would require a thorough design on how to process data from different sources in order to mitigate any consequences on the predicted continuous values for the test set.

In general, it is expected that the federated learning approach can be more beneficial for small to medium-size companies, which would otherwise rely on smaller internal data sets. Nevertheless, our study allowed us to highlight that the federated learning strategy, even with some limitations, can bring value to the drug discovery scenario, bridging the needs of industrial partners to get more data for training ML models without sharing any confidential information.

### 4.2 Conclusions

In this study, we reported our experience as a company joining the Effiris federated learning hackathon organized by Lhasa Limited.

The Effiris federated learning approach was evaluated with help from internal data generated for five targets. It could be shown and confirmed that the federated data have a positive effect on the applicability domain expansion. The performance change of the models with the addition of federated data was generally target dependent.

In order to assess the benefits and drawbacks of the federated learning approach, we compared the SOHN and MLP algorithms implemented in Effiris with internally built ML models, evaluating their performance on a predefined Roche test set. In the majority of cases, the predictive ability of the federated models did not outperform that of the internal models. In fact, in many cases, internal models were demonstrated to be the most reliable option. Nevertheless, federated learning has the potential to allow, in the future, more reliable, stable, and confident predictions on a broader chemical space that could be driven by further updates and model improvements. With this perspective, federated learning can represent a potential new opportunity in the field of drug discovery and safe data and knowledge sharing.

## Supporting Information Available

Composition of the data sets formed by publicly available compounds gathered by Lhasa Limited for each of the end points tackled by Roche in the hackathon; best-performing model for each of the training setups used for the different end points considered in the hackathon; t-SNE plots (PDF)

## Abbreviations

ML: Machine Learning
MCC: Matthews Correlation Coefficient
MAE: Mean Absolute Error
RNN: Recurrent Neural Networks
LSTM: Long–Short-Term Memory
SOHN: Self-Organizing Hypothesis Network
MLP: Multilayer Perceptron
FLuID: Federated Learning using Information Distillation
COX2: Cyclooxygenase-2
GABA_A_: Gamma Aminobutyric Acid Receptor A
hERG: human *Ether-à-go-go*-Related Gene
AChM_1_R: Acetylcholine Muscarinic Receptor 1
5-HT_2B_: 5-Hydroxytryptamine Receptor 2B
t-SNE: t-Distributed Stochastic Neighbor Embedding
GN: Gaussian Naïve-Bayes
SGD: Steepest Gradient Descent Classifier
DT: Decision Tree Classifier
RF: Random Forest Classifier
ET: ExtraTrees Classifier
KNN: K Nearest Neighbors Classifier
AB: AdaBoost Classifier
GB: Gradient Boosting Classifier
SVC: Support Vector Classifier
XG: eXtreme Gradient Boosting Classifier
CV: Cross Validation
AD: Applicability Domain
T: SOHN Teacher Model
HI: SOHN Hybrid Internal Model
HF: SOHN Hybrid Federated Model
PI: MLP Pretrained Model on Public Data
F: MLP Federated Model
RI: MLP Pretrained Model Refined with Internal Data
RF: MLP Federated Model Refined with Internal Data
