# Supplementary material for: Federated Learning in Computational Toxicology: An Industrial Perspective on the Effiris Hackathon
Source: Chem Res Toxicol. 2023 Aug 16;36(9):1503–17. doi: 10.1021/acs.chemrestox.3c00137 (PMC10523574; doi:10.1021/acs.chemrestox.3c00137)
Supplement: Supplementary file 1 — tx3c00137_si_001.pdf [file tx3c00137_si_001.pdf]

Federated Learning in Computational Toxicology: an Industrial Perspective on the Effiris Hackathon

Supplementary Materials

Davide Bassani[1], Alessandro Brigo[1], and Andrea Andrews-Morger[1]*

[1] Pharmaceutical Research & Early Development, Roche Innovation Center Basel, F. Hoffmann-La Roche Ltd., 4070 Basel, Switzerland

* Correspondence: andrea.andrews-morger@roche.com

KEYWORDS: federated, machine, learning, hackathon, Effiris, computational, toxicology, SOHN, FLuID, MLP.

Tables

Target	Database dimension (inactive/active)	Together with all internal data (inactive/active)
$AChM_1R$	2191 (1288/903)	6878 (4862/2016)
$GABA_A$	2433 (1645/788)	6924 (5680/1244)
$5\text{-}HT_{2B}$	1506 (685/821)	5766 (3851/1915)
hERG	8298 (4157/4141)	11139 (5854/5285)
COX2	3228 (1545/1683)	7151 (5169/1982)

Table S1. *Table representing the composition of the datasets formed by public available compounds gathered by Lhasa Limited for each of the endpoints tackled by Roche in the hackathon. On the right column, also the composition of the dataset composed by both Lhasa public and internal Roche training data is reported, coming from the sum of the compounds in the two sets.*

Endpoint	Training dataset	Best-performing model architecture
AChM$_1$R	Roche internal	XGBoost Classifier
AChM$_1$R	MLP Federated labels	SVC
AChM$_1$R	MLP Federated labels + Roche internal data	SVC
AChM$_1$R	MLP FLuID labels	MLP Classifier
AChM$_1$R	SOHN Federated labels	XGBoost Classifier
AChM$_1$R	SOHN Federated labels + Roche internal data	XGBoost Classifier
AChM$_1$R	SOHN FLuID labels	XGBoost Classifier
GABA$_A$	Roche internal	SVC
GABA$_A$	MLP Federated labels	MLP Classifier
GABA$_A$	MLP Federated labels + Roche internal data	MLP Classifier
GABA$_A$	MLP FLuID labels	ExtraTrees Classifier
GABA$_A$	SOHN Federated labels	Gaussian Naïve-Bayes Classifier
GABA$_A$	SOHN Federated labels + Roche internal data	SVC
GABA$_A$	SOHN FLuID labels	XGBoost Classifier
COX-2	Roche internal	SVC
COX-2	MLP Federated labels	MLP Classifier
COX-2	MLP Federated labels + Roche internal data	MLP Classifier
COX-2	MLP FLuID labels	AdaBoost Classifier
COX-2	SOHN Federated labels	SVC
COX-2	SOHN Federated labels + Roche internal data	SVC
COX-2	SOHN FLuID labels	XGBoost Classifier
hERG	Roche internal	K-Neighbors Classifier

hERG	MLP Federated labels	K-Neighbors Classifier
hERG	MLP Federated labels + Roche internal data	K-Neighbors Classifier
hERG	MLP FLuID labels	MLP Classifier
hERG	SOHN Federated labels	ExtraTrees Classifier
hERG	SOHN Federated labels + Roche internal data	K-Neighbors Classifier
hERG	SOHN FLuID labels	Gradient Boosting Classifier
5-HT_{2B}	Roche internal	XGBoost Classifier
5-HT_{2B}	MLP Federated labels	SVC
5-HT_{2B}	MLP Federated labels + Roche internal data	SVC
5-HT_{2B}	MLP FLuID labels	MLP Classifier
5-HT_{2B}	SOHN Federated labels	SVC
5-HT_{2B}	SOHN Federated labels + Roche internal data	SVC
5-HT_{2B}	SOHN FLuID labels	ExtraTrees Classifier

Table S2. *Table representing indicating the best-performing model for each of the training setup used for the different endpoints considered in the hackathon.*

Figures and Figure Legends

Figure S1. t-SNE plots representing the distribution of the chemical space for the $GABA_A$ datasets. Specifically, panel A depicts the reduction of the pool of features composed by the 208 RDkit physicochemical descriptors and the 2048-bits fingerprints to just two dimensions, while on panel B the three-dimensional reduction is reported.

Figure S2. t-SNE plots representing the distribution of the chemical space for the hERG datasets. Specifically, panel A depicts the reduction of the pool of features composed by the 208 RDkit physicochemical descriptors and the 2048-bits fingerprints to just two dimensions, while on panel B the three-dimensional reduction is reported.

Figure S3. t-SNE plots representing the distribution of the chemical space for the COX2 datasets. Specifically, panel A depicts the reduction of the pool of features composed by the 208 RDkit physicochemical descriptors and the 2048-bits fingerprints to just two dimensions, while on panel B the three-dimensional reduction is reported.

Figure S4. t-SNE plots representing the distribution of the chemical space for the 5-HT_{2B} datasets. Specifically, panel A depicts the reduction of the pool of features composed by the 208 RDkit physicochemical descriptors and the 2048-bits fingerprints to just two dimensions, while on panel B the three-dimensional reduction is reported.

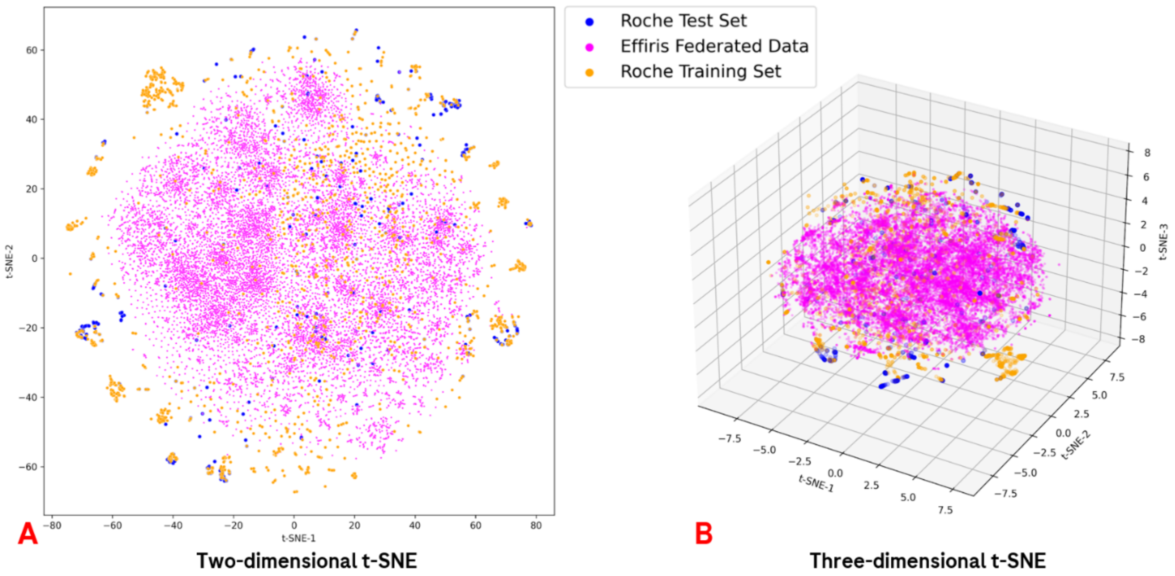

Figure S1.

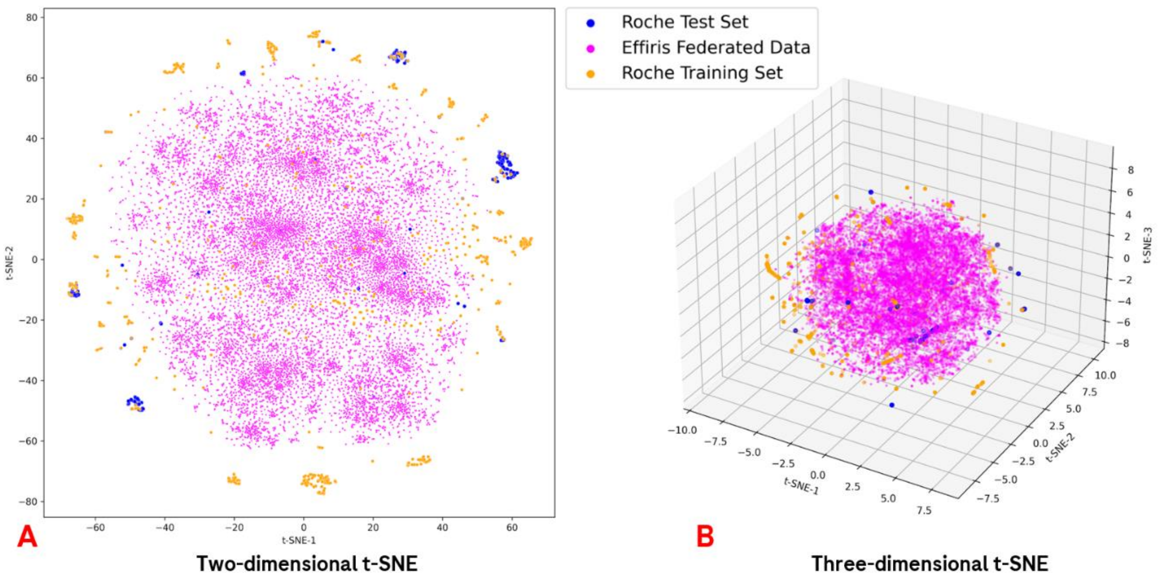

Figure S2.

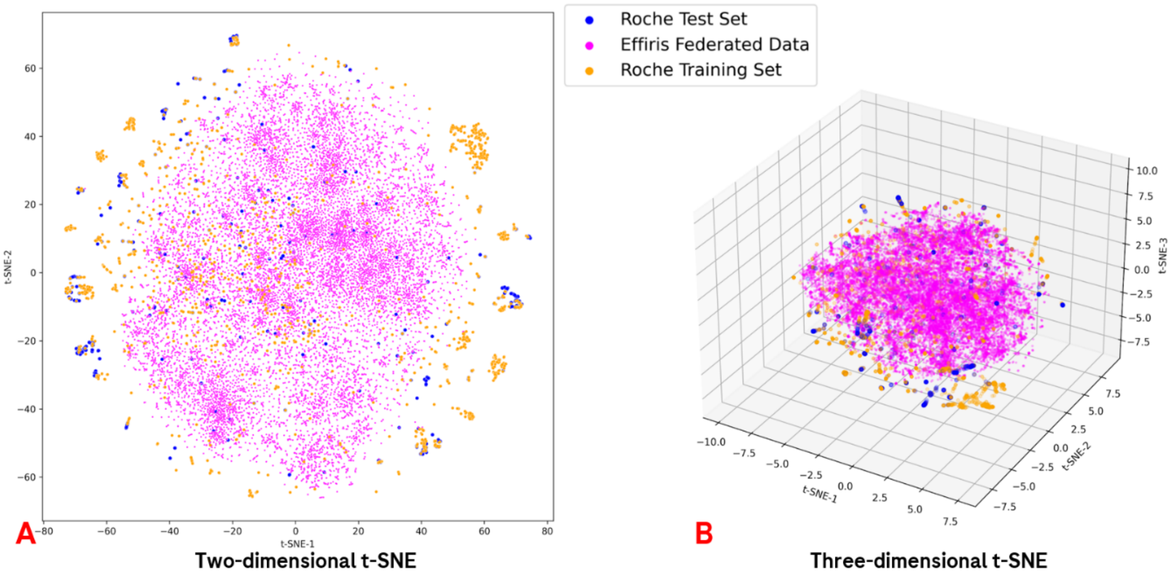

Figure S3.

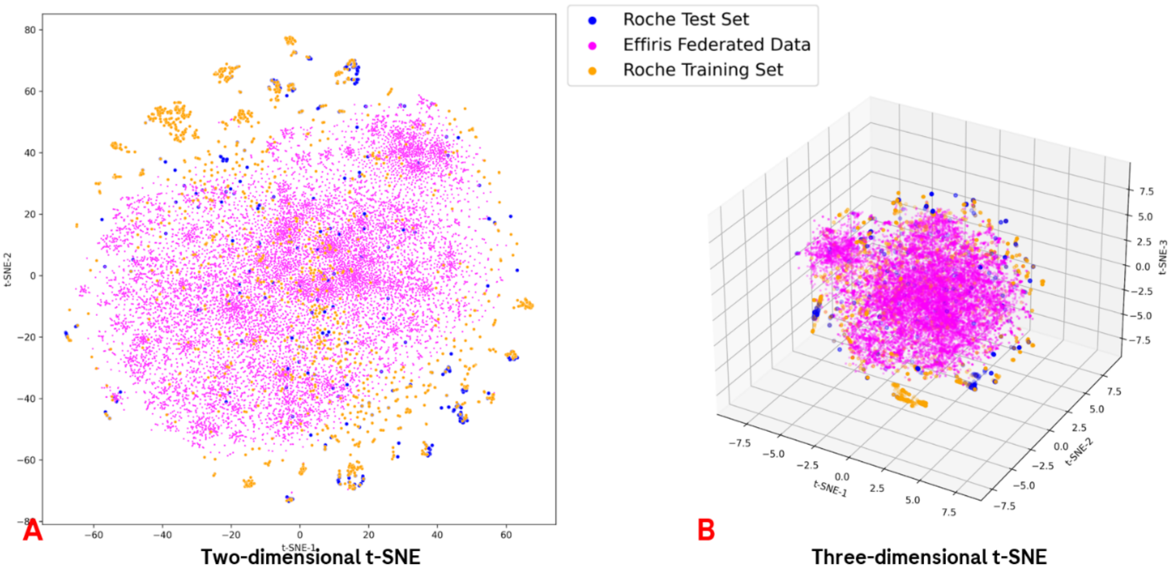

Figure S4.
